# Dietary Inulin Regulated Gut Microbiota and Improved Neonatal Health in a Pregnant Sow Model

**DOI:** 10.3389/fnut.2021.716723

**Published:** 2021-08-09

**Authors:** Hao Li, Longteng Ma, Longlin Zhang, Nian Liu, Zhiqing Li, Fan Zhang, Xiang Liu, Xiaokang Ma

**Affiliations:** College of Animal Science and Technology, Hunan Agricultural University, Changsha, China

**Keywords:** inulin, sow, piglet, health, gut microbiota

## Abstract

This study aimed to investigate the relationship between maternal dietary fiber intake and piglet health. Multiparous sows were randomly assigned to two groups and fed diets without inulin (control group, *n* = 20) or 1.6% inulin (1.6IN group, *n* = 20). The results indicate that 1.6IN prevented the prolonged farrowing duration of sows (*P* < 0.05) and shortened the average piglet birth interval (*P* < 0.1). In addition, 1.6IN decreased the percentage of the piglet born weak and the percentage of the piglet with hyperthermia after birth (*P* < 0.01). Compared with the control group, the 1.6IN group had a lower concentration of urea nitrogen in the colostrum, and also prevented diarrhea, increased litter gain, survival rate, and average daily gain for suckling piglets (*P* < 0.05). Furthermore, 1.6IN decreased the relative abundance of Firmicutes, *Cyanobacteria*, and *Streptococcus*; increased the relative abundance of Bacteroidetes, *Desulfovibrio, Paludibacter, CF231*, and *Prevotella*. Overall, this study showed that maternal fiber nutrition during pregnancy regulated the health of offspring, and the response of the maternal intestinal microbes played an important role in intervening in the phenotype of sows and neonatal piglets.

## Introduction

In the intensive pig industry, sows suffered from both endogenous oxidative stress and exogenous stress induced by environmental and management factors, which led to serious adverse reactions on their offspring, such as prolonged birth intervals, low birth weight, and diarrhea (1). These adverse reactions dramatically increased the risk of non-infectious death in neonatal piglets (2). Fortunately, intestinal microbiota has become an important window for regulating the health of sows and their neonatal piglets because of its close relationship with immunity, metabolism, nutrient digestion, and hormones (3–5).

Feeding functional dietary fiber during pregnancy, especially soluble dietary fiber (SDF), has become a key nutritional strategy for improving reproductive performance in sows, based on its significant regulatory effect on intestinal microbiota (1, 6). As a typical SDF, inulin-type fructans are a mixture of polymers and oligomers, which are composed of fructosyl units linked by β (2 → 1) glycosidic bonds (7). In previous studies, inulin has been proven to increase the abundance of probiotics, such as *Bifidobacterium* and *Lactobacillus*, in the intestine in human or mouse experiments (8–10). Zhou et al. (11) confirmed that inulin inhibited the weight gain of pregnant sows caused by high-fat diets and improved the BMI distribution of newborn piglets (11). The previous study also confirmed that sows fed with inulin increased birth weight and pre-weaning survival for piglets (12); however, it is still necessary to understand the relationship between maternal dietary fiber intake and piglet health.

Therefore, this study aimed to investigate the relationship between maternal dietary fiber intake during late pregnancy and piglet health. Phenotypes of sows and piglets, as well as serum markers and intestinal flora of sows, were analyzed to provide some microbial mechanistic insights into the application of inulin to a typical gestation diet of sows for improving neonatal health and performance.

## Materials and Methods

### Ethics Statement

The protocol of this study was approved by the Institutional Animal Care and Use Committee of College of Animal Science and Technology, Hunan Agricultural University (Changsha, China) and was conducted in accordance with the National Institutes of Health (Changsha, China) guidelines for the care and use of experimental animals (No. 43321809). The inulin was provided by Sensus (RG Roosendaal, The Netherlands) with 90% purity.

### Experimental Animals, Diets, and Sample Collection

**Figure 1:**
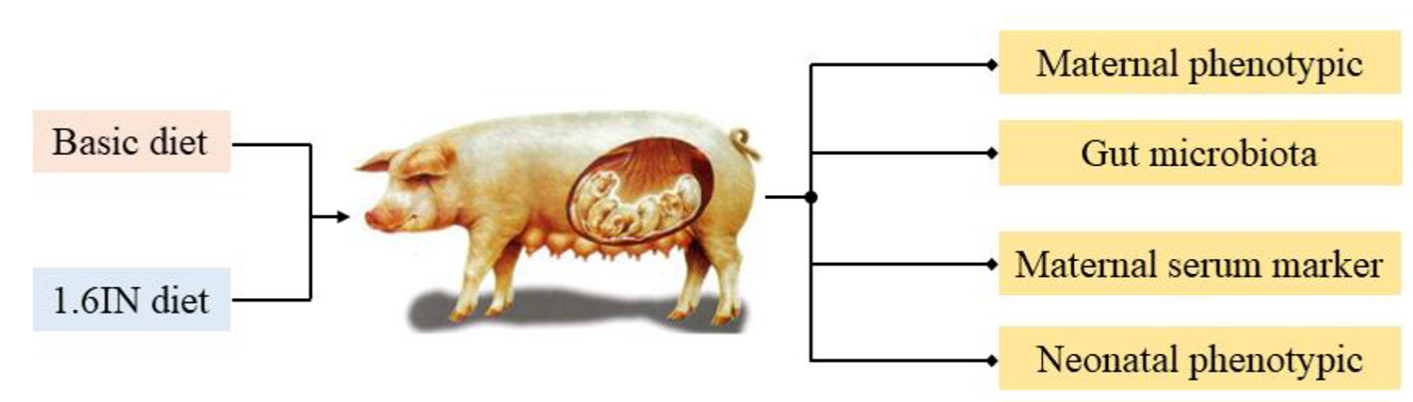
Experimental design of this study.

A total of 40 Landrace × Yorkshire second parity sows were selected for this experiment. All the sows were fed with the same standard diet from mating to gestation d80. Then, they were allocated to one of two treatments randomly as a single factorial experimental design after balancing their backfat thickness and body weight. The sows were fed with two different diets: a basic diet based on corn and soybean meal (control group, *n* = 20), and a diet that included 1.6% inulin (1.6IN group, *n* = 20). During gestation from d80 to d109, the sows in each group were fed a daily ration of 3.3 kg dry matter (DM) with their respective diets containing 11.94 ± 0.03 MJ ME/kg. Then, the sows were moved from the gestation pens to the farrowing rooms on day 109 ± 1 of gestation and kept in individual stalls (2.2 × 0.75 m). The sows were offered 3 kg DM of the same lactation diet containing 13.7 MJ ME/kg DM (Table 1) and were fed two times a day before farrowing. From the 1st day postpartum until weaning, the sows of both treatments were fed *ad libitum* with the same standard lactation diet (Table 1). All the sows had free access to water during the whole experimental period. The experimental design of this study was shown in Figure 1.

**Table 1:** Feedstuff ingredients and nutrient composition of experimental diets.

**Items**	**Control**	**1.6IN**	**Lactation diet**
**Ingredients, %**			
Corn	56.00	56.00	65.00
Soybean meal	8.00	8.00	20.00
Fermented soybean meal	5.00	5.00	5.00
Soybean oil	1.00	1.00	2.00
DDGS^a^	2.00	2.00	0.00
Soybean hull	16.00	15.20	0.00
Rice bran	8.00	7.20	3.60
Inulin^b^	0.00	1.60	0.00
Salt	0.45	0.45	0.50
L-Lys	0.00	0.00	0.20
D-Met	0.00	0.00	0.10
Dicalcium phosphate	1.18	1.18	1.20
Calcium carbonate	1.37	1.37	1.40
Mineral-vitamin pre-mix^c^	1.00	1.00	1.00
Total	100.00	100.00	100.00
**Nutrient composition**			
ME of DM, MJ/kg	11.97	11.93	12.94
Crude protein, %	13.98	13.95	17.23
Crude fiber, %	8.18	8.09	2.63
Calcium, %	0.92	0.92	0.88
Phosphorus, %	0.54	0.51	0.58
Total dietary fiber, %	26.61	27.51	16.46

a *DDGS, distillers dried grains with soluble*.

b *Inulin contains 94% DM, 89.8% inulin, 3.2% monosaccharide, <0.2% crude protein and ash, average monomeric units = 13*.

c *Provided per kg of diet: Cu, 10 mg (as CuSO_4_·5H_2_O); Fe, 110 mg as ferrous sulfate; Mn, 35 mg (as MnO_2_); Zn, 65 mg as zinc sulfate; I, 0.6 mg as potassium iodide; Se, 0.3 mg as selenium selenite; vitamin A, 7,200 IU; vitamin D3, 1,500 IU; vitamin E, 30 mg; vitamin K, 1.2 mg; 1 mg, thiamin; 2 mg, riboflavin; 1 mg, pyridoxine; and 0.015 mg, cobalamin*.

Colostrum samples (30 ml) were collected from the third, fourth, and fifth pairs of mammary glands of sows (eight sows per diet group) on the farrowing day. Then, the colostrum samples were immediately frozen at −20°C until further analysis. Fresh fecal samples were collected from the sows (eight sows per group) on day 109 ± 1 of gestation and day 18 of lactation. Then, the fecal samples were stored at −80°C until further analysis.

### Performance Measurement

The birth time of each piglet was recorded, which was used to calculate farrowing duration and average piglet birth interval (APBI). After farrowing, the rectal temperature of each piglet was recorded with a digital thermometer (Xiaomi Co. Ltd, Beijing, China, with a display resolution of 0.01 and ± 0.1°C accuracy) and weighed before suckling. Piglets weighing <800 g were recorded as born intrauterine growth retardation (IUGR); otherwise, they were regarded as born effective.

Cross-fostering was kept within diet treatments to adjust litter size to about 12.86 ± 1.2 piglets per sow and average body weight to about 1.8 ± 0.7 kg per litter within 48 h after parturition. During lactation, mortality of each piglet was recorded, and the occurrence of diarrhea was visually assessed and evaluated by individual scoring of the consistency of the feces from 9.00 a.m. to 4.00 p.m. each day by trained observers blind to the treatments according to the method of Marquardt et al. (13). The diarrhea rate (%) was calculated as [(the total number of piglets with diarrhea within a treatment)/(total number of experimental piglets × total observational days)] × 100. At weaning (lactation d18), the number of piglets was recorded to calculate the weaning survival rate, and the litter weight was also recorded to calculate the litter gain and the average daily gain (ADG).

### Analysis of Colostrum Composition

The colostrum samples of sows in each group were separately analyzed for the concentrations of fat, protein, lactose, urea nitrogen (UN), and total DM using Milko-Scan FT 120 (Foss Electric, Hillerford, Denmark). Somatic cell count (SCC) was measured using FOSS MATIC 5000 (Foss Analytical A/S, Hillerod, Denmark).

### DNA Extraction, PCR Amplification, Library Preparation, and Sequencing

DNA was extracted from fecal samples of sows using a Stool DNA Isolation Kit (Tiangen Biotech Co., Ltd., Beijing, China). The V4 hypervariable region of the bacterial 16S rRNA gene was amplified by PCR, where the forward primer was 550F: 5′-GTGCCAGCMGCCGCGGTAA-3′ and the reverse primer was 806R: 5′-GGACTACHVGGGTWTCTAAT-3′. For each fecal sample, a 10-digit barcode sequence was added to the 5′ end of the forward and reverse primers. The sequences were clustered into operational taxonomic units (OTUs) at a similarity level of 97% to generate rarefaction curves and to calculate the richness and diversity indices. OTUs representing <0.005% of the population were removed, and taxonomy was assigned using the Ribosomal Database Project (RDP) classifier. The relative abundance of each OTU was counted at different taxonomic levels. OTU-level alpha diversity indices were calculated using the OTU table in QIIME. β-diversity was assessed by principal component analysis (PCoA) based on the Bray–Curtis distance. Bioinformatics analysis was mainly performed using QIIME (v1.7.0) and R packages (v3.2.0).

### Analysis of Fecal Short-Chain Fatty Acids

The concentration of SCFAs in feces was analyzed using a gas chromatographic method, as described by Bosch et al. (14). Briefly, approximately 1.5 g of feces was first homogenized in 1.5 ml of deionized water. The samples were centrifuged at 15,000 × *g* at 4°C for 10 min. Supernatants (1 ml each) were then acidified with 25% metaphosphoric acid at a 1:5 ratio (1 volume of acid for 5 volumes of the sample) for 30 min on ice. The sample was injected into a GC 2010 series gas chromatograph (Shimadzu, Kyoto, Japan) equipped with a CP-Wax 52 CB column 30 m × 0.53 mm i.d. (Chrompack, Rotterdam, Netherlands). The injector and detector temperatures were 75 and 280°C, respectively. Total SCFAs were determined as the sum of analyzed acetate, propionate, and butyrate. All procedures were performed in triplicate.

### Analysis of Serum Marker in Sows

Venous blood from the ear margin of the sow on the day of parturition was used to separate serum. Serum markers, such as malondialdehyde (MDA), total antioxidant capacity (TAOC), superoxide dismutase (SOD), glutathione peroxidase (GSH-Px), lipopolysaccharide (LPS), and lactate were determined using commercial kits by following the instructions of the manufacturer (Nanjing Jiancheng Co. Ltd., Nanjing, China).

### Statistical Analysis

Litter gain, survival rate, piglet ADG, diarrhea rate, serum marker, SCFA composition, α-diversities index, and relative abundance were tested for normality and were then analyzed by an unpaired *t*-test (SPSS 21.0, IBM, Armonk, NY, United States), using each sow as an experimental unit. Data were presented as means ± SEM except that confidence limits were given in brackets instead of SEM values for data of relative abundance at phylum. A chi-square test was performed to analyze the percentage of sows that had a prolonged farrowing duration or prolonged average piglet birth interval and to analyze the percentage of piglets born weak or with hyperthermia after birth. Statistical significance was declared when *P* < 0.05.

## Results

### Farrowing Duration of Sows and Average Piglet Birth Interval

**Figure 2:**
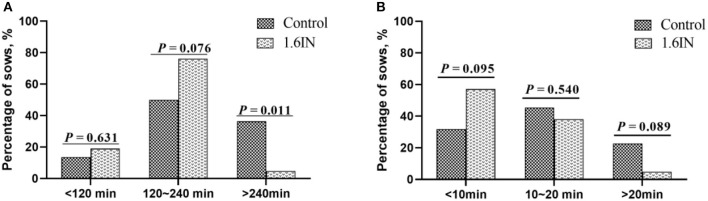
Distribution of **(A)** farrowing duration and **(B)** average piglet birth interval of sows. *N* = 20 for the control group and 1.6IN group. Significance was analyzed by chi-squared test.

The results of dietary inulin on farrowing duration and APBI are shown in Figure 2. Compared to the control group, 1.6IN decreased the percentage of sows whose farrowing duration was longer than 240 min (*P* = 0.011) and trend to decreased percentage of sows whose APBI was longer than 20 min (*P* = 0.089). In addition, 1.6IN also increased the percentage of sows whose APBI was shorter than 10 min on a trend (*P* = 0.095).

### Performance of the Piglet

**Figure 3:**
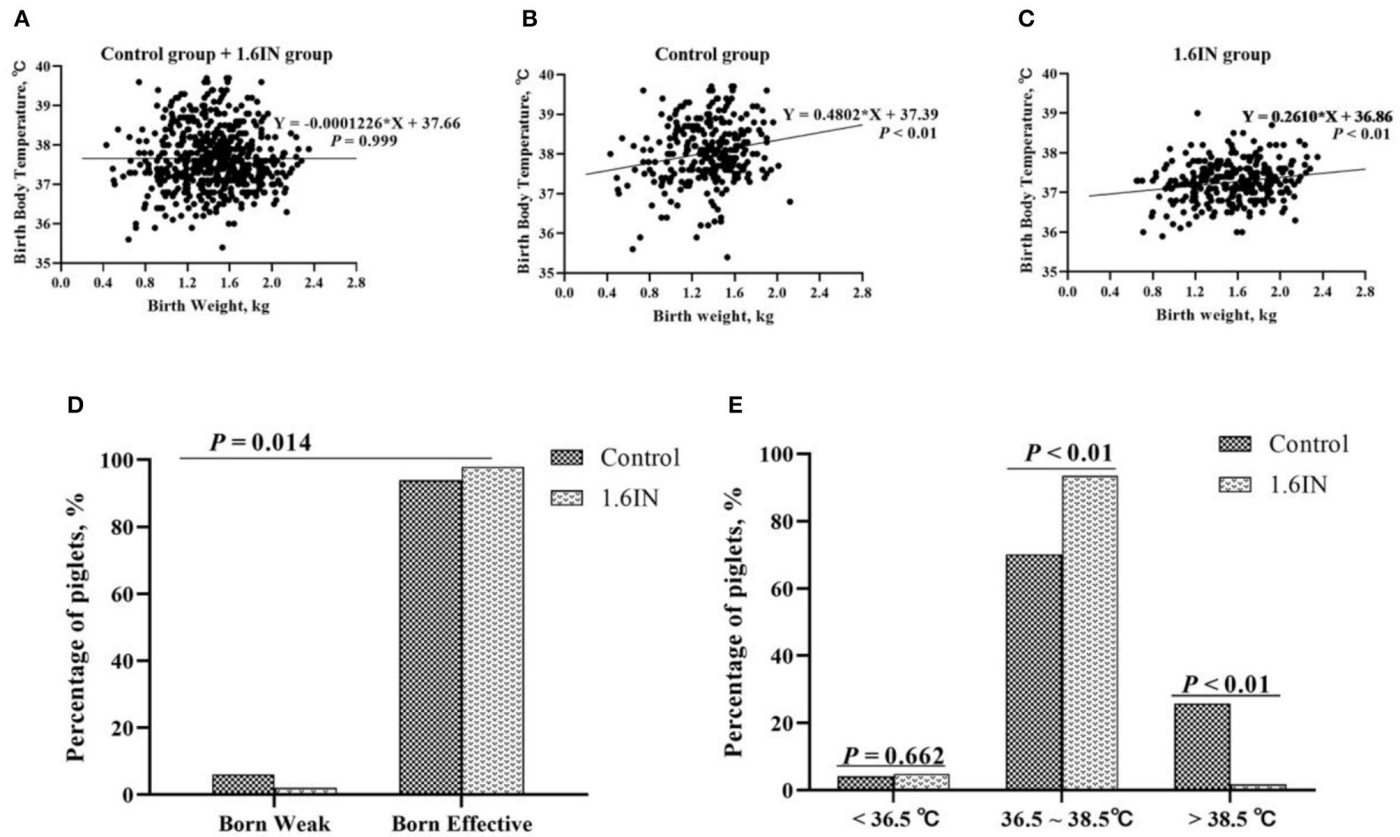
**(A–C)** Relationship between birth weight and birth body temperature of piglets and **(D,E)** effects of dietary inulin on the birth weight and birth body temperature of newborn piglets. *N* = 295 and 291 for the control group and 1.6IN group, respectively. A Chi-square test was conducted to judge whether **(D)** low birth weight improved or **(E)** distribution of birth body temperature of newborn piglets changed.

As shown in Figure 3, the birth weight and birth body temperature of a total of 586 piglets from two groups were recorded. When the two groups were analyzed together, there was no significant relationship between the body temperature and weight of newborn piglets; however, when the two groups were analyzed separately, there was a significant linear relationship between piglet body temperature and weight. The control group has a higher slope and intercept, which suggests that the piglets of the control group may have a higher average body temperature, which is more pronounced in high birth weight piglets. In addition, it could be observed that the birth body temperature of piglets was mainly enriched at 36.5–38.5°C. Therefore, piglets with body temperatures lower than 36.5°C and higher than 38.5°C are judged as hyperthermia and hypothermia, respectively. A Chi-square test was conducted to confirm whether dietary inulin improved IUGR or prevented hyperthermia or hypothermia in newborn piglets (Figure 3). The results show that 1.6IN decreased the percentage of the piglet in IUGR (*P* < 0.05) and the percentage of the piglet in hyperthermia (*P* < 0.01).

**Figure 4:**
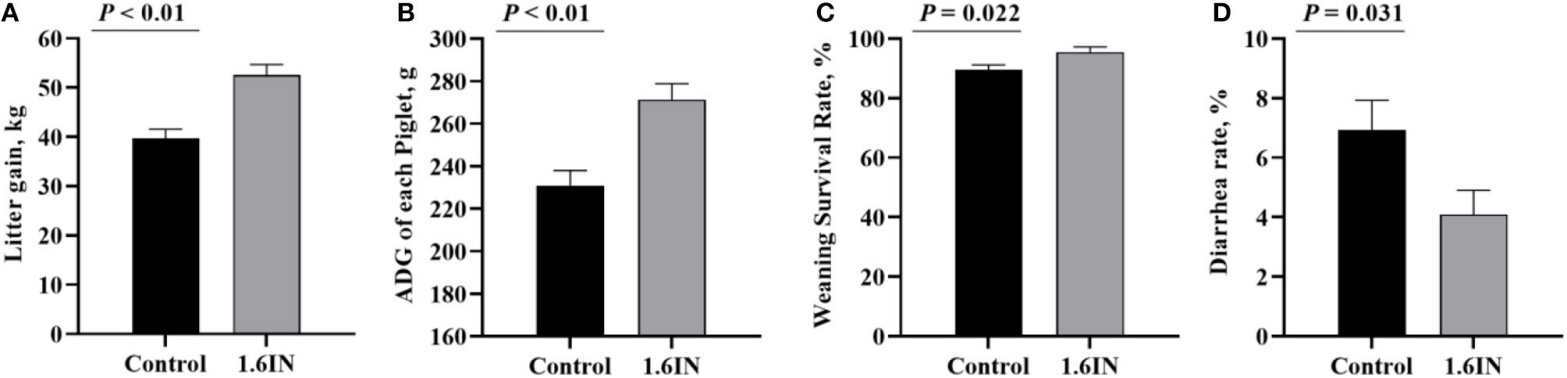
Effects of dietary inulin on the piglet performance during lactation. **(A)** litter gain at weaning; **(B)** average daily gain (ADG) of each piglet; **(C)** survival rate at weaning; **(D)** diarrhea rate during lactation. Significance was analyzed by an unpaired *t*-test.

The piglet performance from cross-fostering to weaning is presented in Figure 4. Compared with those in the control group, the piglets in the 1.6IN group had higher litter gain and survival rate at weaning (*P* < 0.01), and 1.6IN also increased piglet ADG and decreased diarrhea rate during lactation (*P* < 0.05).

### Colostrum Composition

The results of dietary inulin on colostrum composition are shown in Table 2. The colostrum from the 1.6IN group had a lower concentration of UN compared with the control group (*P* < 0.05); however, there was no difference in fat, protein, lactose, DM, and SCC between the two groups (*P* > 0.05).

**Table 2:** The effect of dietary inulin on colostrum composition of sows.

**Items**	**Control**	**1.6IN**	***P*-value**
Fat, %	5.35 ± 1.01	4.48 ± 0.43	0.448
Protein, %	17.55 ± 1.08	17.72 ± 0.78	0.901
Lactose, %	4.24 ± 0.19	4.13 ± 0.16	0.667
DM, %	35.31 ± 1.00	34.49 ± 0.91	0.556
UN, mmol/L	66.60 ± 5.69	51.9 ± 2.87	0.042
SCC, L	3,997.00 ± 2,581.00	795.00 ± 259.00	0.271

### Serum Marker of Sows

**Figure 5:**
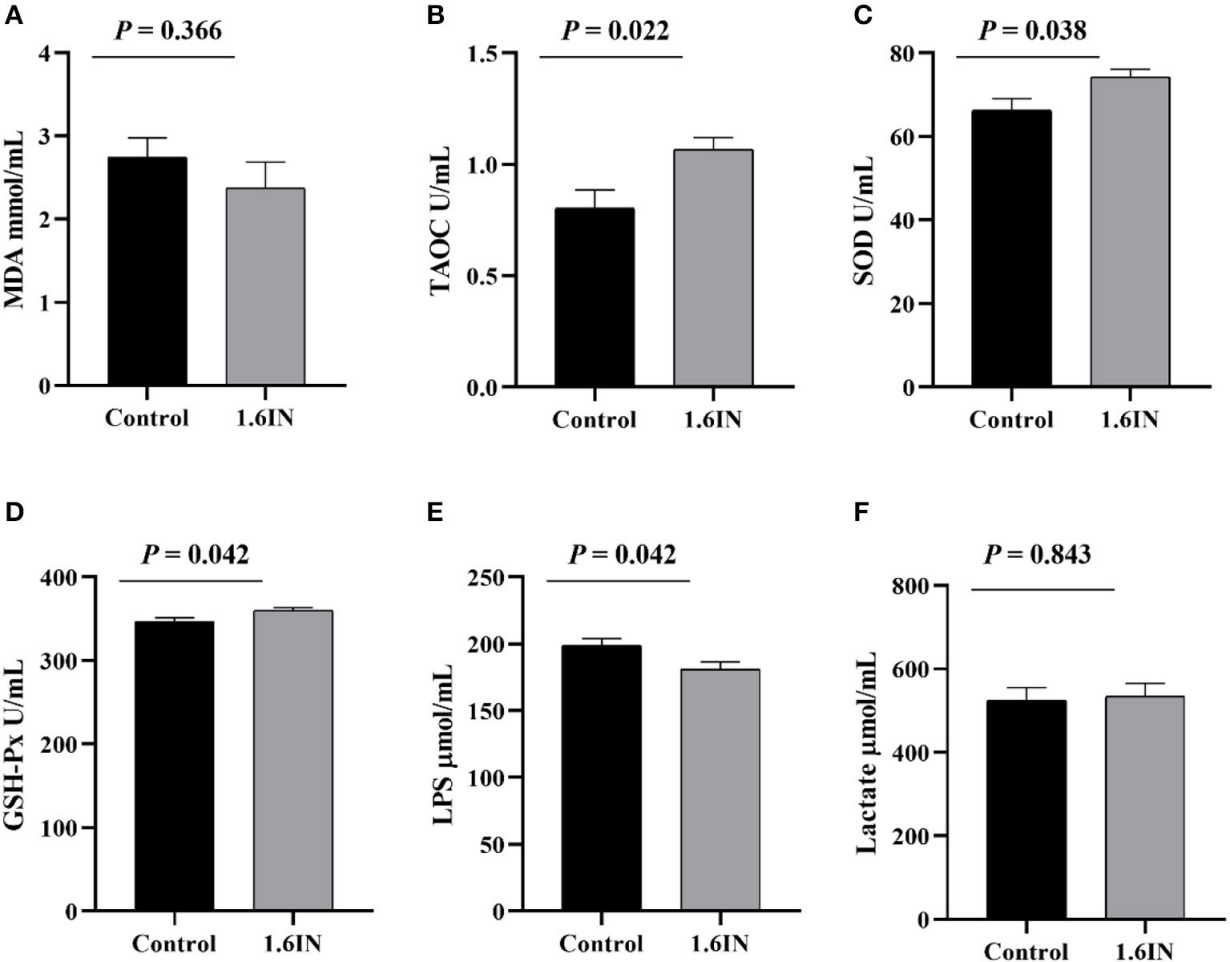
Effects of dietary inulin on the serum markers of sows **(A)** malondialdehyde, MDA; **(B)** total antioxidant capacity, TAOC; **(C)** superoxide dismutase, SOD; **(D)** glutathione peroxidase, GSH-Px; **(E)** lipopolysaccharide, LPS; **(F)** lactate. Significance was analyzed by an unpaired *t*-test.

The results of dietary inulin on a serum marker of sows are shown in Figure 5. The colostrum from the 1.6IN group had higher levels of TAOC, SOD, and GSH-Px compared with the control group (*P* < 0.05); however, there was no difference in MDA and lactate between the two groups (*P* >0.05).

### OTU Partition and Microbial Diversity Analysis

**Figure 6:**
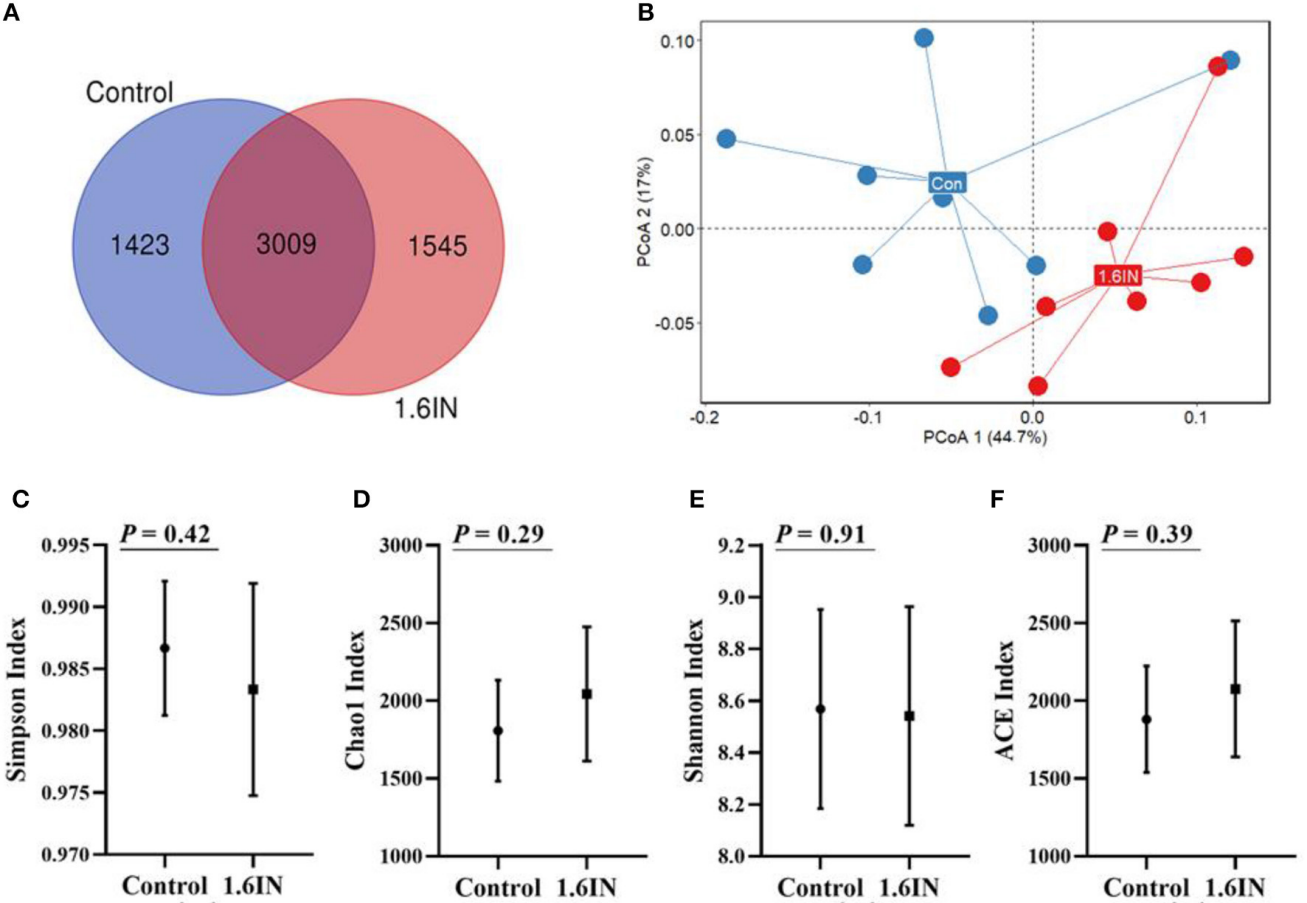
**(A)** Venn diagram exhibits the shared and unique operational taxonomic units (OTUs) between two groups and **(B)** principal component analysis (PCoA) based on genus level, each point represented one sample, blue points from the control group and red points from the 1.6IN group. **(C)** Simpson index; **(D)** Chao1 index; **(E)** Shannon; **(F)** ACE index. Significance was analyzed by an unpaired *t*-test.

There were means of 4,432 and 4,554 OTUs from the control group and the 1.6IN group, respectively, and there were 3,009 common OTUs between the two groups (Figure 6A). There was no difference in α-diversity, such as Shannon index, Chao1 index, Simpson index, and ACE index between the two groups (Figure 6), indicating that bacterial richness was not affected by dietary inulin. The microbial communities in all the samples were analyzed and compared by the PCoA (Figure 6B). The first two components accounted for 61.7% variation; however, no great variation could be observed between the control group and the 1.6IN group (*P* = 0.09).

### Taxonomic Composition Analysis

**Figure 7:**
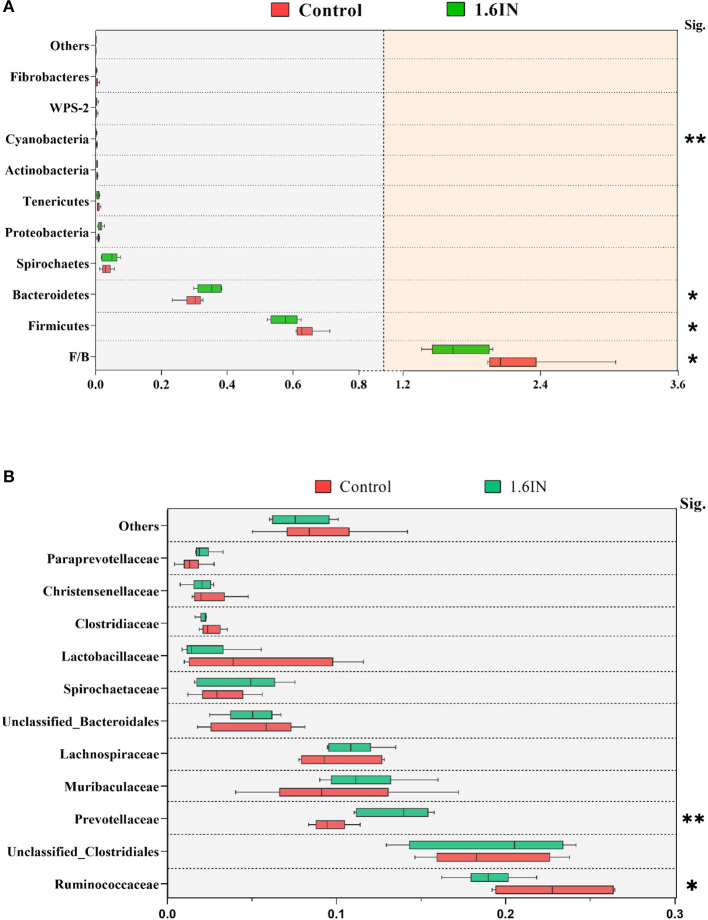
Taxonomy composition of the bacterial communities at **(A)** the phylum level and **(B)** the family level (average relative abundance >0.001 at least one group). Significance was analyzed by an unpaired *t*-test. *P < 0.05; ** *P* < 0.01.

The results of phylum distribution are shown in Figure 7. Taxonomic assignment of the OTU identified 15 phyla in the fecal samples of sows in this study. Nine phyla (average relative abundances >0.1% in at least one group) were chosen for significance analyses, suggesting that the top two phyla, Firmicutes and Bacteroidetes, were dominant in the fecal samples of sows with >90% total relative abundance. Compared with the control group, 1.6IN decreased the relative abundance of Firmicutes, *Cyanobacteria*, and the ratio of Firmicutes/Bacteroidetes (*P* < 0.05) and increased the relative abundance of Bacteroidetes (*P* < 0.05). At the family level, 1.6IN increased the relative abundance of *Prevotellaceae* (*P* < 0.01) but increased the relative abundance of *Ruminococcaceae* (*P* < 0.05).

**Figure 8:**
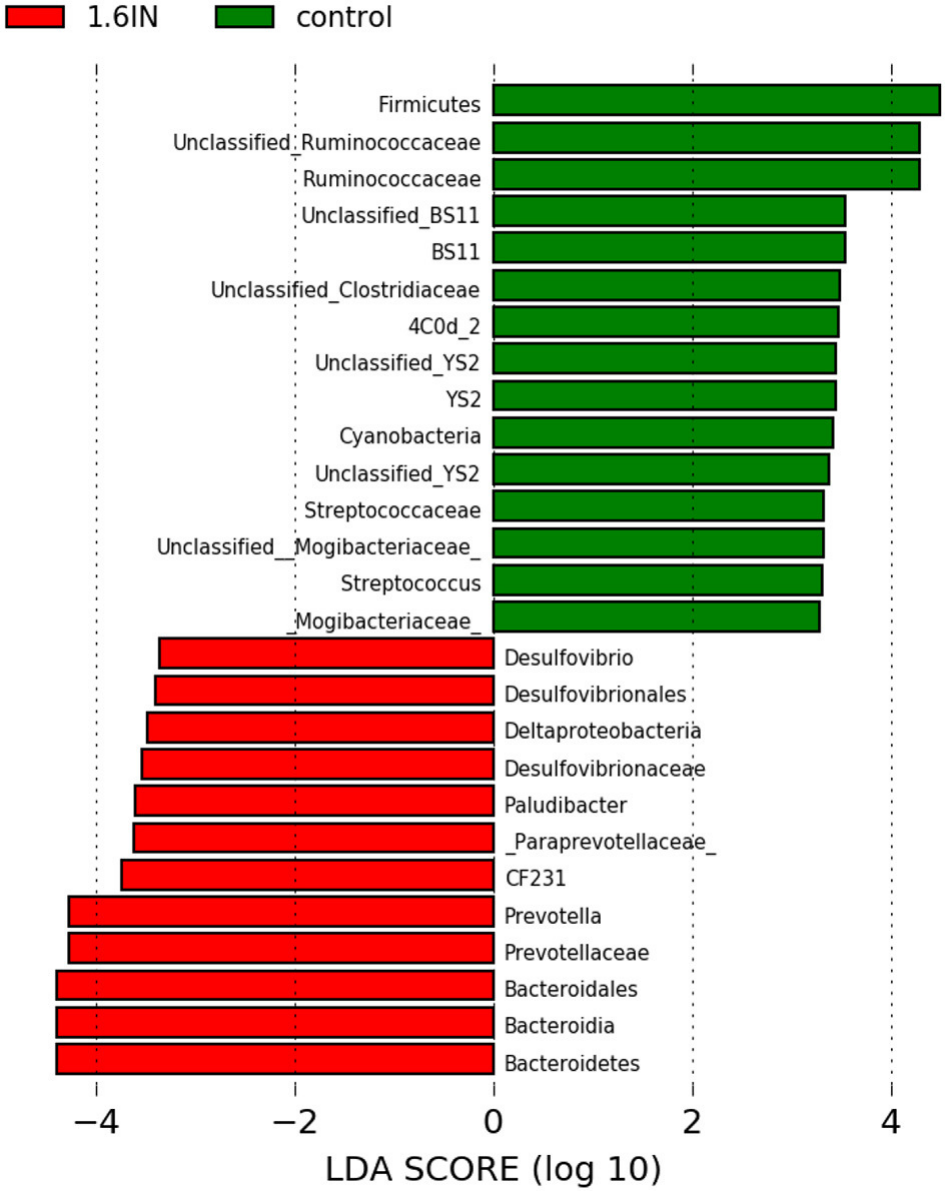
LefSE analysis of colonic microbiota between two dietary groups. LDA scores are calculated for characteristics at the OTU level, and if the value for the LDA score is >3, it means there is a significant difference.

To identify the specific bacterial taxa among the groups, we compared the fecal microbiota by using LEFSE analysis. The results showed 25 different OTUs between the two groups, 10 OTUs were highly abundant in the 1.6IN group and 15 OTUs in the control group (Figure 8). At the family level, a great abundance of *Ruminococcaceae, BS11, YS02, Streptococcaceae, Mogibacteriaceae* in the control group, and a great abundance of *Desulfovibrionaceae* and *Paraprevotellaceae* in the 1.6IN group was found. At the genus level, a great abundance of *CF231, Paludibacter, Prevotella*, and *Desulfovibrio* in the 1.6IN group and *Streptococcus* in the control group was observed.

### Fecal SCFA Composition

The results of microbial metabolite SCFAs are shown in Table 3. There was no difference in the concentration of acetate, propionate, butyrate, and total SCFAs (*P* > 0.05); however, 1.6IN increased the ratio of acetate in the total SCFAs significantly compared with the control group (*P* < 0.05).

**Table 3:** The effect of dietary inulin on SCFA composition in the feces of sows.

**Items**	**Control**	**1.6IN**	***P*-value**
**Concentration, umol/g**			
Acetate	93.54 ± 5.38	99.76 ± 2.38	0.315
Propionate	31.31 ± 1.76	29.97 ± 1.35	0.561
Butyrate	14.12 ± 1.51	13.14 ± 0.83	0.585
Total SCFAs	138.96 ± 6.94	142.88 ± 3.55	0.627
**Ratio, %**			
Acetate	67.2 ± 0.86	69.84 ± 0.70	0.038
Propionate	22.73 ± 1.42	20.97 ± 0.75	0.299
Butyrate	10.07 ± 0.76	9.18 ± 0.48	0.349

### Correlations Between Gut Microbiota and Colostrum Composition, Newborn Body Index of Piglets, and Serum Marker of Sows

**Figure 9:**
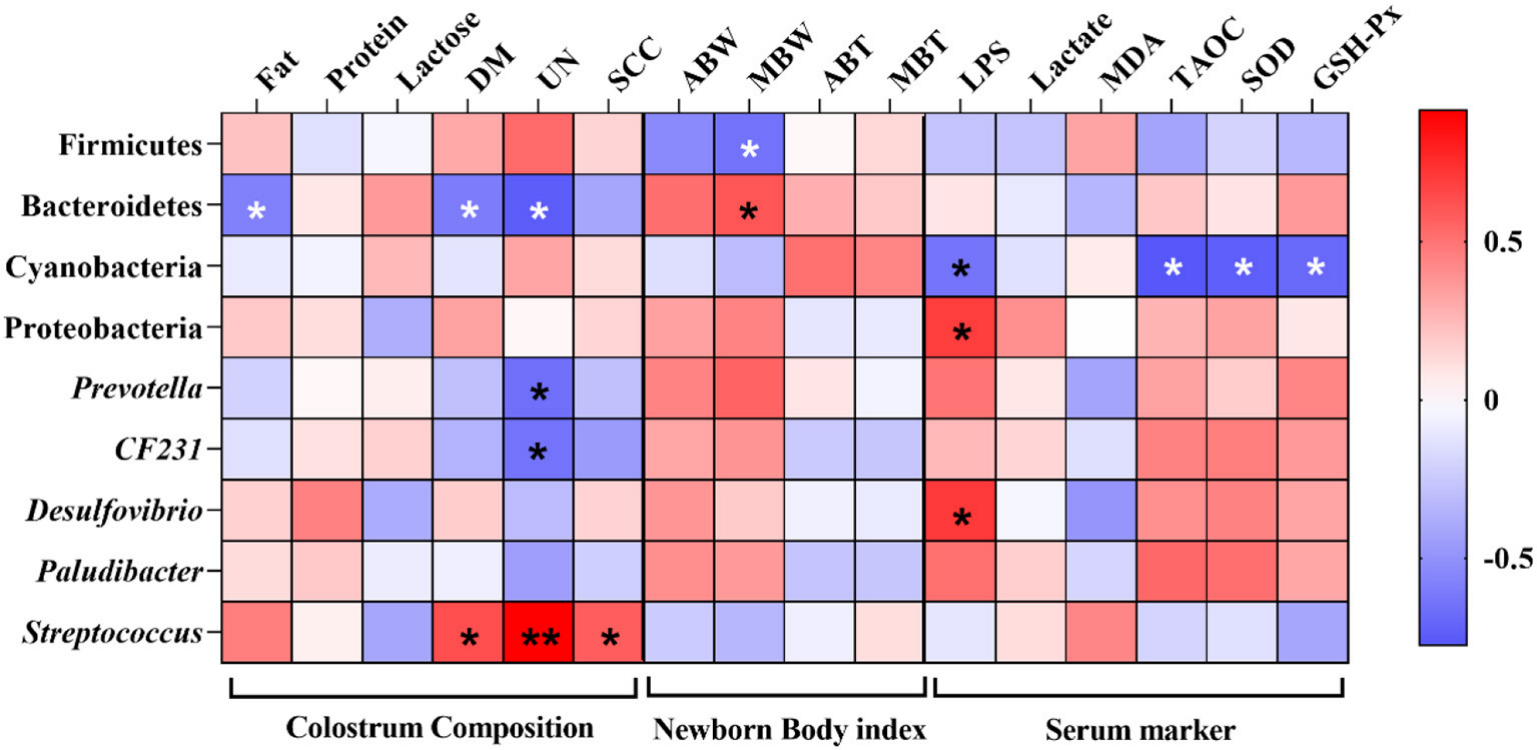
Heatmap of the Spearman's *r* correlations between the gut microbiota significantly modified by different diets treatment and colostrum composition, newborn body index of piglets, and serum marker of sows. ABW, average body weight; MBW, median body weight; ABT, average body temperature; MBT, median body temperature. Significance and correlation coefficient was analyzed by Spearman's correlation analysis. *P < 0.05; ** *P* < 0.01.

A Spearman correlation analysis was performed to evaluate the potential link between alterations in gut microbiota composition and colostrum composition, newborn body index of piglets, and serum marker of sows (Figure 9). The concentration of fat, DM, and UN was negatively correlated with the phylum Bacteroidetes (*P* < 0.05). In addition, the UN concentration was also negatively correlated with the genus *Prevotella* and *CF231* (*P* < 0.05), and the concentration of UN and SCC was positively correlated with the genus *Streptococcus* (*P* < 0.05). Firmicutes and Bacteroidetes were negatively and positively correlated with the median body weight (MBW) of newborn piglets (*P* < 0.05). Furthermore, lipopolysaccharide (LPS) was negatively correlated with *Cyanobacteria* and positively correlated with *Proteobacteria* and *Desulfovibrio* (*P* < 0.05), respectively, and *Cyanobacteria* also was negatively correlated with TAOC, SOD, and GSH-Px (*P* < 0.05).

## Discussion

Because of specific physiological conditions and feeding procedures, pregnant sows are exposed to a series of inevitable problems, such as weight gain during pregnancy, constipation, and prolonged farrowing duration (15). Affected by the above physiological problems from their mothers, newborn piglets often die of low birth weight and poor viability before weaning (2, 15). Birth weight depends on nutritional status and placental transport function during late gestation, while viability is closely related to birth weight and farrowing duration, and may be reflected in the body temperature (16, 17).

Previous studies have suggested that dietary fiber was conducive to shortening the farrowing duration and improved piglet birth weight (12, 18, 19). In this study, 1.6%, the dose with the best improvement effect, was selected as the inulin dosage from the previous study (12). The results of this study showed that 1.6IN reduced the percentage of sows whose farrowing duration was >240 min and that APBI was >20 min. 1.6IN also reduced the percentage of IUGR in the piglet and improved the survival rate before weaning. The reason for these results may be that inulin improved the antioxidant capacity and energy metabolism for sows, which were consistent with the results of previous studies (5, 12, 20).

The body temperature of the piglet during birth and the diarrhea rate before weaning were selected as indicators for judging the health of the piglet. It has been reported that body temperature during birth as an indicator affects survival and growth performance due to which unnormal body temperature is considered to be associated with increased mortality (21, 22). Hypothermia indicated lack of suckling capacity and subsequent growth retardation, whereas hyperthermia may be caused by inflammation, and it means that piglets consume too much-stored energy and oxygen to provide heat, and it may lead to decreased digestive enzyme activity, and cause diarrhea and reduced growth rate (23, 24). In this study, there was no difference between the two groups in the percentage of piglets whose body temperature was lower than 36.5°C, whereas the percentage of piglets with body temperature higher than 38.5°C was significantly reduced in the 1.6IN group. In addition, 1.6IN also reduced the rate of diarrhea and increased the ADG of the piglets, indicating that the preventive effect of 1.6IN on hyperthermia helped to relieve diarrhea of suckling piglets. It was reported that an improvement in intake of the maternal SDF on the antioxidant capacity and the inflammation in the colon of piglets were observed via regulation of the community of gut microbiota, which could explain the results of body temperature in piglets reasonably (12).

Breast milk is the most important source of nutrients, energy, and immunologically active substances for piglets before weaning. In previous studies, dietary fiber in the late gestation could affect the colostrum composition for sows, so this study determined the concentration of fat, protein, lactose, DM, UN, and SCC in colostrum (18, 25). The results showed that 1.6IN did not affect fat, protein, lactose, and DM in colostrum, which meant that there was no difference in the nutritional content of colostrum between the two groups; however, six samples from the control group had elevated UN (*P* < 0.05) and SCC concentrations (*P* > 0.05), which are important indexes for judging milk quality or mastitis (26, 27). The diarrhea rate of piglets in the control group was also significantly higher than that in the 1.6IN group, which also may be caused by low-quality milk from the inflamed breast.

Increasing research focuses on the interactions among diet, gut microbiota, and the host (4, 28). The results of this study have shown that Firmicutes and Bacteroides dominate at the phylum level, which can reach more than 90% relative abundance of the total gut microbiota of sow. The ratio of Firmicutes to Bacteroides (F/B) has been judged to be an important index for affecting the energy metabolism of mammals, which was usually related to energy deposition in humans, mice, and pigs (29, 30). 1.6IN reduced the F/B ratio, affecting the median body weight (MBW) of newborn piglets from correlation results, indicating that the sows fed diet with dietary inulin deposited less energy under the same calorie intake, and the undeposited part may be allocated to the development of the fetus, which was potentially causal with the reduction in the rate of low birth weight (11).

Furthermore, the control group also had a higher relative abundance of *Streptococcaceae* and *Mogibacteriaceae*, which contained lots of common conditional pathogens (31, 32). In particular, *Streptococcus*, one of the core strains in milk, and parasitizing in the breast potentially, was identified as a higher relative abundant species in the control group (33). In previous studies, *Streptococcus* has usually shown a high correlation with mastitis of cows (34). In this study, the relative abundance of *Streptococcus* also showed a positive correlation with the concentration of UN and SCC in colostrum, which suggested the potential connection between *Streptococcus* and sow mastitis. Therefore, the reduction of *Streptococcus* may be the key reason for 1.6IN to reduce UN and SCC in colostrum; however, the results did not confirm whether the *Streptococcus* translocated into the sow breast from the intestines, which required further research.

The physiological status of sows largely determined the health of offspring piglets. Six blood markers that reflect the health status of sows were tested in this study, wherein MDA, SOD, TAOC, and GSH-Px reflected antioxidant capacity (12), LPS reflected intestinal barrier function (35), and lactate reflected the degree of anaerobic respiration of sows during farrowing (36). The results showed that inulin increased the concentrations of SOD, TAOC, and GSH-Px in the serum of sows, suggesting an improvement in antioxidant capacity, which was consistent with previous studies; however, inulin also increased the concentration of LPS, suggesting a reduction in intestinal barrier function of sows.

Two phyla, closely related to serum markers, deserved our attention. Proteobacteria include many common opportunistic pathogens, such as *Escherichia coli* and *Desulfovibrio* (37). *Desulfovibrio* was increased in the 1.6IN group. It can reduce the sulfur-containing substance to produce hydrogen sulfide that irritates mucosa, causing decreased barrier function, and increased serum LPS concentration (38, 39); however, the tolerance of pig immune cells to LPS stimulation has been previously reported (40), and we have not identified a significant stress response in sows and their offspring. Therefore, we have reservations about the negative effects of LPS in sows. *Cyanobacteria*, containing bacteria that produced natural toxins, were found to be significantly higher in the control group (41). A characteristic increase in intestinal *Cyanobacteria* on progeroid mice has been reported by previous studies (42). The results of correlation analysis also showed that *Cyanobacteria* were negatively correlated with TAOC, SOD, and glutathione peroxidase (GSH-Px). Therefore, we speculated that dietary inulin may improve the antioxidant capacity of sows by downregulating the relative abundance of *Cyanobacteria* in the gut microbiota.

In research on dietary fiber, SCFAs were thought of as a “bridge” in the diet–gut microbiome-host metabolism axis (43). Acetate (C2), propionate (C3), and butyrate (C4) are the most abundant, representing more than 90% of the SCFAs present in the colon. The majority of SCFAs are absorbed by colonic epithelial cells, and only 5–10% is excreted in the feces. SCFAs can regulate fat synthesis and cholesterol in the liver, and stabilize blood glucose by triggering glucagon secretion and increasing satiety (44). SCFAs regulate intestinal inflammation in sows and inhibit fat deposition in sows, which has the potential to be a beneficial intervention for positive pregnancy outcomes (5). Results align with those obtained by Marquardt et al. (13) and Zhou et al. (11) who did not detect any significant effects of inulin inclusion on the concentration of SCFA and its constituents in feces of sows during late gestation; however, the feces sample from 1.6IN had a higher acetate ratio in total SCFAs. The relative abundance of acetate-producing bacterium *Prevotella* and *CF231* was also higher in the 1.6IN group, which provided a reasonable explanation for the results of acetate ratio (29, 45).

## Conclusion

This study verified the beneficial effect of inulin as a functional fiber in the nutrition of sows in late pregnancy, not only in the reproductive performance of sows but also in the survival of newborn piglets. Overall, this study showed that maternal fiber nutrition during pregnancy regulated the health of offspring, and the response of the maternal intestinal microbes played an important role in intervening in the phenotype of sows and neonatal piglets.

## Data Availability Statement

The datasets presented in this study can be found in online repositories. The names of the repository/repositories and accession number(s) can be found at: https://www.ncbi.nlm.nih.gov/sra/?term=PRJNA736251.

## Ethics Statement

The animal study was reviewed and approved by Institution Animal Care and Use Committee of college of Animal Science and Technology, Hunan Agricultural University (No.43321809) (Changsha, China).

## Author Contributions

HL and XM designed the research. XM provided the funding. HL, LM, and XL conducted the research. HL and NL analyzed the data. HL mainly wrote the manuscript. LZ, ZL, and FZ edited the manuscript. All authors contributed to the article and approved the submitted version.

## Conflict of Interest

The authors declare that the research was conducted in the absence of any commercial or financial relationships that could be construed as a potential conflict of interest.

## Publisher's Note

All claims expressed in this article are solely those of the authors and do not necessarily represent those of their affiliated organizations, or those of the publisher, the editors and the reviewers. Any product that may be evaluated in this article, or claim that may be made by its manufacturer, is not guaranteed or endorsed by the publisher.
